# Class Room Talks, No. 4

**Published:** 1888-05

**Authors:** 


### CLASS-ROOM TALKS. No. 4.

(FROM LECTURES BY PROFESSOR J. T. KENT, M. D.)

The proving of Silicea superinduced in the provers a tendency to, and a deposit of, tubercular and calcareous masses in the lungs, in the abdomen, in the lymphatic glands, in the brain and spinal cord, in old cicatrices; tubercular deposits in any part of the body, such as are found in tuberculosis-phthisis, tabes-mesenterica, etc.; friable, calcareous deposits, found in arthritic and pulmonary calculi; in calcareous deposits in the glands, or calcareous deposits in any part of the body, and it causes these deposits to suppurate.

Great danger to patients of these peculiar diatheses may follow a prescription of Silicea, especially if the disease is of long standing and well advanced and the remedy is repeated. The danger lies in that tendency in Silicea—as in Sulphur—to excite the suppurative process, especially about foreign bodies; the tubercle or calculi sustaining such a relation to the system, Silicea seems able to excite in the vital force an effort at expulsion, which, if the deposit were deep and extensive, would in all probability be of fatal result through destruction of the parenchyma of the lungs, if the disease is there located. In a very small deposit there might be a chance of suppuration and healing without destroying so much of the lung-tissue as would endanger life. Hence, we see that Silicea is a dangerous remedy in cases of tubercular deposits in any of the deeper and more vital organs because of this suppurative tendency. Silicea is a wonderful remedy in tubercular-meningitis, basilar-meningitis when indicated and not repeated, but if you do not *know* and observe well the disease in all its modalities, you will kill instead of cure your patients; you will consider that which *is* really an improvement a worse condition, and interference at this time means death. You may have prescribed for a child that has been roused from the state of coma and general paresis, but he begins to roll and toss about the bed, screaming out in his agony, until he drives the friends and the doctor nearly frantic. The parents and friends will all beg you to find him relief; they will implore you for opiates, for anything, to relieve the agony, and if you are not very firm, with the firmness that knowledge gives, you will lose him yet. You must seat yourself gravely with these people, explain to them carefully the terrible alternative and the prospect of recovery if you allow the remedy its full and perfect work. You must show them that the distress is caused by the most violent formication in the skin, with biting, stinging, and itching, almost unbearable, owing to the renewed activities of the nerves. Leave it alone for two or three days, and for your forbearance you will be blessed with his return to health.

Silicea has another marked characteristic. Nature shows a tendency to encyst foreign bodies, as shot, balls, etc., which received into the body accidentally remain imbedded and finally become encysted with a protective plasma. They are thus often prevented from creating disturbance to the economy for many years. Should a patient, having suffered such an experience, come to you, no matter if Silicea were clearly indicated, beware how you give it if the foreign body is deeply located or supposedly near a vital organ, as the remedy may, if repeated, excite the suppurative process, creating long, tubular sinuses, and before the work of expulsion can be accomplished you will have a *funeral*. Never give Silicea when you have reason to think there is a deeply imbedded bullet in a patient.

You will often be surprised at the opening of an old cicatrix, with discharge of the tuberculous or calcareous deposit, showing again the common tendency to suppuration and expulsion of foreign bodies. What signification could we find in the text of the *Materia Medica*, where it reads, "Expulsion of foreign bodies," were it not for this tendency to expulsion by suppuration?

A Silicea cold will begin with continued sneezing, dryness of the mucous membrane, headache, vertigo, coming up through the spine and neck and spreading over the head. This condition gives way to an excessive flow of thick, bloody mucus, or mucus streaked with blood, from the nose, which gradually changes to a yellow, pus-like, long-continued catarrhal discharge.

With an abscess in the mammary gland a good homœopath is expected to deal so effectually that his patient passes the different stages without discomfort or pain, no matter how large or uncomfortable-*looking* the abscess may be.

Right here let me tell you, don't put on a poultice, for it will spoil the action of the homœopathic drug. Why? Well, I usually want a reason for what I do—and a pretty sound one, too—and I will give you one which should be strong enough even for the physiological humbugs of this latter day. Administer a *homœopathic* remedy, and what do you expect to find as the law of its action? What do you expect it to do? Why, you expect it to *immediately* begin the process of removing from the congested and inflamed parts the superabundant blood. It does so, and the tension is relieved, the pain is gone, and only such part of the blood as has been effused into the tissue remains to be washed out by the suppurative process, or resorbed into the system. Now, what is the action when the poultice is applied? Why, it is diametrically opposed to this process—calling the blood to the part and eliminating the effusion by a forcing process.

In the matter of prescribing for a tape-worm—*don't do it!* A tape-worm will not live in a healthy stomach, and when you have cured the patient the worm will take his departure. It is both possible and probable that a stomach and intestine so diseased as to grow and feed a tape-worm may be restored to a state of health that will no longer furnish him the necessary nutrition. He begins the search for it rather than starve, and ultimately finds himself in new quarters and is very quickly dispatched. *Don't doctor the worm.* I am always sorry when I know he is present, as I keep thinking of his wormship and desire to give him a big dose and put him out. I can do it.

What is a homœopathic prescription? Does it make a prescription homœopathic to use potentized remedies, to carry minute medicated sugar pellets, to take them from a peculiar case, to use a high potency or a low potency, to use a small quantity of a drug instead of a large, to graduate from a homœopathic college, to call one's self homœopathic and then use

Quinine and Morphia to cover our ignorance? I tell you, *no!* These medicines I carry in my case are not homœopathic; the name on the sign, be it in ever so big letters, is not homœopathic; the medicines used are still drugs, and improperly administered may do even more evil than the same drug in the crude. What is it, then? Prescribing according to the exact *law of Similars.* That, and that alone, means a homœopathic prescription; that, and that alone, means success; that, and that alone, will make your name in connection with Homœopathy, in all time to come, honored above all men, as one learned in the science of the art of healing, and give you a satisfaction than which there is *no greater.* S. L. G. L.

